# RAS/Mitogen-Activated Protein Kinase Signaling Pathway in Testicular Germ Cell Tumors

**DOI:** 10.3390/life14030327

**Published:** 2024-02-29

**Authors:** Angelo Onorato, Eugenia Guida, Ambra Colopi, Susanna Dolci, Paola Grimaldi

**Affiliations:** Department of Biomedicine and Prevention, University of Rome Tor Vergata, 00133 Rome, Italy; onorato.ange@gmail.com (A.O.); eugenia.guida@uniroma2.it (E.G.); ambracolopi@yahoo.it (A.C.); dolci@uniroma2.it (S.D.)

**Keywords:** testicular germ cell tumors (TGCTs), KIT, MAPK, RAS, cancer, PGC, epigenetics

## Abstract

Germ cell tumors (GCTs) are relatively rare tumors. However, they are the most diagnosed malignancies occurring in the testis among men aged between 15 and 40 years. Despite high aneuploidy and a paucity of somatic mutations, several genomic and transcriptomic assays have identified a few significantly mutated somatic genes, primarily KIT and K-RAS. The receptor Tyrosine Kinase (RTK) pathway and the downstream related Mitogen-Activated Protein Kinase (MAPK) cascades are crucial signal transduction pathways that preside over various cellular processes, including proliferation, differentiation, apoptosis, and responses to stressors. They are well described in solid malignancies, where many of the involved factors are used as prognostic molecular markers or targets for precision therapy. This narrative review focused, in the first part, on PGCs’ survival/proliferation and differentiation and on the genetic and epigenetic factors involved in the pathogenesis of testicular germ cell tumors (TGCTs) and, in the second part, on the most recent investigations about the KIT-RAS pathway in TGCTs and in other cancers, highlighting the efforts that are being made to identify targetable markers for precision medicine approaches.

## 1. Introduction

### 1.1. Germ Cell Origin and Prenatal Development

Germ cells are the unique cells that transmit their genome to the new organism through the process of fertilization. Their genetic material is kept in a genetically stable and epigenetically reprogrammable state to allow the specification and the development of new gametes that will be specified when the embryo will form. The specification of the germline in mammalian embryos is inductive and involves a complex transcription factor core regulatory network in accordance with a combination of growth factors. The genetically determined transcriptional program controlling the specification and determination of the germline in mammals has been the subject of extensive research during the past fifteen years. The work conducted on mouse models has yielded much of the current knowledge about mammalian germline development. However, significant species-specific variations in the transcription factor network between mouse and human primordial germ cell (mPGC and hPGC) development have been identified [1,2].

Recent advances in pluripotent stem cell technology have made it possible to overcome the ethical and technical obstacles in studying germline specification in humans by creating surrogate cell culture models. In fact, human primordial germ cell-like cells (hPGCLCs) derived from pluripotent stem cells have researchers allowed to investigate this process in vitro [3].

By exploiting in vitro derivation of hPGCLCs, it has been demonstrated that a lineage-primed progenitor expressing the transcription factor TFAP2A is most likely the source of hPGCs [3]. Under the influence of the growth factor BMP4, epiblast cells with a transitional pluripotent state—which shares characteristics with pre- (ground state naive pluripotency) and post-implantation (primed state) epiblasts—in the implanted blastocyst are selected for TFAP2A expression on the 11th day post conception (dpc) [4]. TFAP2A+ progenitors that express the mesodermal/endodermal transcription factors GATA3, EOMES, and BRACHYURY (also defined as T) transiently possess the ability to differentiate toward both the germ cell and the somatic cell fates. Extended exposure to BMP4 in the progenitors activates the expression of TFAP2C, which is located upstream of BLIMP1 (PRDM1) to control SOX17 expression and germ cell fate determination [3]. Fully specified SOX17/NANOS3 expressing PGCs require then the action of additional factors, such as PRDM14, that cooperates with TFAP2C and PRDM1, to upregulate and maintain germ pluripotency [2].

Following specification, PGCs reach the gonadal anlage by migrating through the hindgut and the dorsal mesentery [5]. While PGCs’ translocation into the gut is an active mechanism in the mouse, it may be passive in the human embryo [5]. It has recently been demonstrated that several PGCs are present in the human embryo at approximately 35 dpc. They have been identified along Schwann cells and autonomic nerve fibers from the dorsal mesentery to the gonad, where they appear to be transported by nerve fibers [6]. A surge in Stem Cell Factor/Kit Ligand (SCF/KL) expression by the surrounding cells is essential to guide germ cells during their migration [6]. Late PGCs that remain in the midline structures (dorsal mesentery) or mistake the correct migratory route should undergo cell death by apoptotic pathway [7]. The growth factors that regulate the survival and/or proliferation of mPGCs in vitro have been shown to be active also on hPGCs [8].

In particular, the Kit/KL (Kit Ligand) system, largely conserved in mammals, regulates PGCs’ growth both in mouse and human PGCs and loss of function mutations at the White Spotting (W) and Steel loci (Sl), which encode the Kit receptor and its ligand (KL), respectively, and leads to germ cell loss [9]. Interestingly, both mouse and human PGCs are prone to be reprogrammed in vitro, giving rise to pluripotent mouse and human embryonic germ cells (mEGCs and hEGCs, respectively), when cultured in the presence of a variety of compounds and growth factors, including LIF, bFGF, and KL [8,10,11]. The ability of PGCs to be reprogrammed is shared by both sexes during the migratory period, but only male germ cells retain such ability for a few more times after gonadal colonization [12]. mEGCs, similarly to embryonic stem cells (ESCs), undergo malignant transformation when transplanted in mice, giving rise to teratomas (TEs) with embryonal carcinoma components (ECs) [13], indicating that PGC latent pluripotency can lead to tumorigenesis in vivo.

### 1.2. Testicular Germ Cell Tumors (TGCTs)

**Figure 1:**
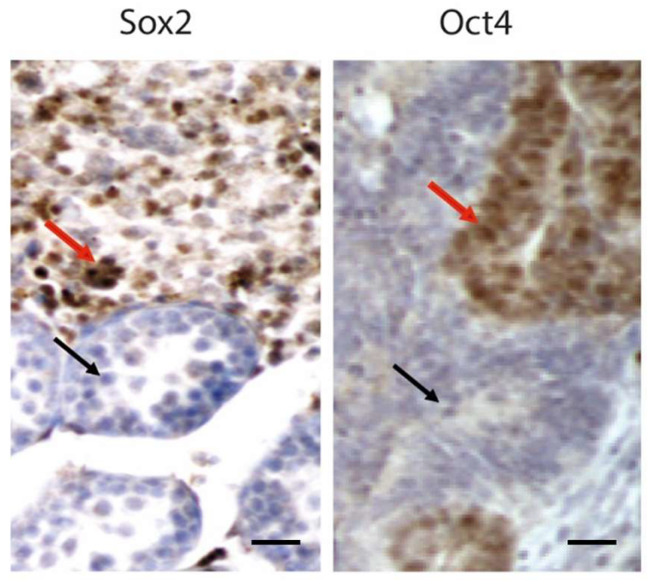
BRafV600E EC disrupted gonadal architecture of a 20 days post-partum mouse testis. Red arrow points to Sox2 and Oct4 positive teratocarcinoma cells. Black arrow points to a normal tubule. Methods and details are found in [20]. Scale bar: 25 µm.

TGCTs are the most common malignancy in European young adult males [14]. Human TGCTs have recently been divided into two categories: germ cell neoplasia in situ (GCNIS, type I), which arises from a pre-existing in situ lesion, and tumors that develop independently from GCNIS (type II) [15,16]. The GCT class unrelated to GCNIS is rare, self-limiting, and typical of elderly men [16]. It includes spermatocytic tumors (STs), which are thought to originate from spermatogonia or spermatocytes, pre-pubertal yolk sac tumors (YSTs,) pre-pubertal teratomas (TEs), and mixed pre-pubertal TEs and YSTs [17]. TGCTs originating from GCNIS occur after puberty in young males [18,19] and can be classified into two groups on the basis of their morphological, immunohistochemical, and molecular evidences: pure classic seminomas (SEs) and non-seminomatous germ cell tumors (NSEs) [16,20]. SEs are less aggressive than non-SEs, which include tumors with different malignancy grades and histological components such as embryonal carcinoma (EC) (Figure 1), trophoblastic tumors (TTs), post-pubertal YSTs, post-pubertal TEs, and teratomas with somatic type malignancy.

It is still unknown what biological factors led to the initial malignant transformation of precursor cells, or gonocytes into GCNIS cells. Most likely, the first transformation takes place in utero during the early stages of the germline’s development in embryonic germ cells [21]. Potential mechanisms that induce germ cell neoplastic transformation during gametogenesis include defective epigenetic remodeling, uncontrolled proliferation, and errors in apoptosis or meiotic commitment [22]. Studies have demonstrated the important role of the MAPK pathway in controlling spermatogonia proliferation [23,24] and in driving male and female mouse teratocarcinomas from late PGCs [21]. However, it is believed that TGCTs arise from a combination of variables and that the growth of tumors is largely influenced by both hereditary and environmental factors [25].

### 1.3. Genetics of TGCTs

Given that TGCTs have among the greatest heritabilities of any cancer, genetic factors may play a more significant role in TGCT development than in other cancer types.

Aneuploidy as whole-chromosome or whole-arm DNA imbalance is a hallmark of human cancers, and aneuploidy is nearly universal in GCTs [26,27]. Somatic genomic profiling studies have identified gain of the short arm of chromosome 12, typically as an isochromosome (i12p), as pathognomonic for TGCTs being present in more than 80% of cases. However, the gene(s) on 12p critical to GCTs have not yet been defined [26].

The small number of large families has limited the search by genetic linkage analysis for familial TGCT susceptibility genes. Until today, only one moderate-penetrance predisposition gene, checkpoint kinase 2 (*CHEK2*), a tumor suppressor gene involved in DNA double-strand break repair, cell cycle regulation, and cellular apoptosis, has been identified [28,29]. Studies including the whole genome have found 78 low-to-moderate-risk single nucleotide polymorphisms, which make up 44% of the risk in families [30]. A number of these genes encode proteins that are engaged in biological processes that are important to the susceptibility to TGCT. These pathways include those involved in male germ cell specification and migration, sex determination and maturation, and regulation of mitosis [30]. The results from mouse models support the direct involvement of multiple target genes in the development of TGCT or TGCT-related disorders. For instance, deletion variants at the Steel locus on the murine 129/Sv background are linked to an increased incidence of TGCT; on the murine 129/Sv background, loss of Dmrt1, which is essential for determining sex and maintaining the male somatic niche, results in an over 90% incidence of testicular Tes [31,32].

### 1.4. Epigenetics of TGCTs

During germline development, male germline cells go through chromatin remodeling mechanisms that result in the production of a distinct epigenome that is different from that of their somatic counterparts. Epigenetic mechanisms that include DNA methylation, posttranslational modifications of histones, and noncoding RNA (miRNA) occur more frequently than genetic mutations and are important for regulating gene expression during male germ cell development. The first epigenetic reprogramming in male germ cells takes place in utero in developing PGCs. They undergo rapid genome-wide erasure of original DNA methylation, including the genomic imprints that are necessary for the development of two types of germ cells accordingly to the sex of the child. When PGCs first differentiate in the mouse embryo at embryonic day (E)6.5, DNA demethylation begins early in these cells. It almost finishes after gonadal colonization at around E13.5 [33] and less than 10% of the total DNA is methylated at this stage of embryogenesis [34]. The DNA methylation status of hPGCs is mostly equivalent to that of mice at similar developmental stages, indicating that these reprogramming dynamics have evolved to be conserved in these two species. Thus, similarly to mPGCs, global erasure of DNA methylation in hPGCs is apparently completed by Wk10-11, around the time of sex determination [35]. Most of DNA re-methylation is initiated before birth, in mitotically arrested prospermatogonia [36]. DNMT3 family members play an essential role in germ cell de novo DNA methylation [37]. Histone modifications occur in mPGCs after the first week of development (for a review see [38]) and after birth during the spermatogenetic process in spermatocytes with a global enrichment in H3K4me3 at the leptotene stage of prophase [39].

Altered epigenetic modifications (e.g., DNA methylation/demethylation, histone modifications, and non-coding RNAs) have been identified in TGCTs [40,41] with distinct genome-wide DNA methylation patterns in different types of TGCTs. For example, it has been demonstrated that the genomes of GCNIS, gonadoblastoma, and seminomas are substantially unmethylated, similar to the genomes of fetal gonocytes, even though they express DNMT1 and, to a lesser degree, DNMT3B and 3L [42,43].

Instead, in non-seminomatous germ cell tumors (NSGCTs), a hypermethylation pattern has been identified [44]. While the methylome of tumors from non-embryonal carcinomas mimics that of extraembryonic lineages, that of ECs is similar to that of ESCs [45,46]. Smiraglia et al. have proposed a model where SEs arise from GCNIS cells derived from PGCs which have undergone global demethylation, while non-seminomas resulted from GCNIS cells that have passed through the de novo methylation [47].

The methylation status of specific genes could be considered as a potential biomarker for TGCTs. For example, the DNA repair genes MGMT, RASSF1A, and BRCA1, and the transcriptional repressor gene HIC1, were frequently methylated in the NSGCTs.

Notably, a splice variant of human RAS effector homologue, encoded by RASSF1A, interacts with the XPA protein and inhibits the proliferation of cells [48]. Promoter methylation has been demonstrated to inactivate RASSF1A in a range of tumor types, including TGCT [49,50].

Histone modifications associated with chromatin structure repression like H3K9me2 and H3K27me3 have been found to be expressed at low levels in GCNIS cells in contrast with H3K4me1, H3K4me2/3, H3K9ac, and a histone variant H2A.Z, which are associated with permissive chromatin structure.

Lastly, it has been discovered that miRNAs are important in TGCTs and offer new potential targets for treatment. Among them, the miR-371–373 cluster and the miR-302 cluster are found overexpressed in TGCTs. Members of the miR-371–373 cluster are the prevalent miRNAs in human embryonic stem cells and are involved in self-renewal processes and in maintaining the pluripotency status of ESCs [51].

The miR-302 cluster plays a critical role in the regulation of the cell cycle in embryonic and pluripotent stem cells. High expression of miR-302s in TGCTs suggests that they act as oncogenes in TGCTs. It is interesting to note that miR-302s has been demonstrated to increase the production of survivin, inhibiting apoptosis, and enhance the expression of SPRY4 and the activation of the MAPK/ERK pathway [52].

### 1.5. MAPK Signaling Pathway in Cancer

The MAPK cascade is a key signaling pathway involved in the transduction of extracellular signals to cellular responses like proliferation, differentiation, apoptosis, and stress responses. In mammalian cells, four leading MAPK families have been characterized: extracellular signal-regulated kinases (ERK1 and ERK2), Jun N-terminal kinases (JNK1, JNK2, and JNK3), p38 kinase, and ERK5 [53,54]. The JNK and p38 MAPK pathways are mainly related to cell stress and apoptosis, while the ERK1/2 and ERK5 MAPK signaling pathway is preferably linked to cell proliferation and differentiation [53,55,56,57,58]. A dual phosphorylation event on MAPK threonine and tyrosine residues activates the phosphorylation of targets on serine and threonine residues within a consensus PXT/SP motif (where the X residue depends on the different MAPK) [59,60].

Activation of the MAPK pathway is based upon sequential activation of several layers of protein kinases (three to five) known as MAPK kinase kinase kinase (MAP4K), MAPK kinase kinase (MAP3K), MAPK kinase (MAPKK), MAPK, and MAPK-activated protein kinases (MAPKAPK). The first three central layers are thought to constitute an important core unit. while the last two layers have been found in some cascades and can differ between cells and stimuli [53].

Dysregulated MAPK signaling is involved in diverse human diseases, primarily cancer [61,62,63,64,65,66,67], and it is generally caused by activating mutations in the receptors or in the downstream signaling effectors that activate the pathway in the absence of appropriate stimuli. Correspondingly, constitutive receptor tyrosine kinase (RTK) pathway activity is prevalent in many cancer types, mainly due to activating mutations in the constituents of the RAS-RAF-MEK axis [68,69].

Among all MAPK signal transduction pathways, the RAS/RAF/MAPKK (MEK)/ERK pathway is the most important signaling cascade and plays a crucial role in the survival and development of tumor cells [53]. Perturbation in the RAS/ERK pathway represents a primer for the development of several cancer types [70,71]. As reported by several authors, the frequency of mutations in the *RAS* gene (mainly *K-RAS*) is about 30% of all cancer types [72] and is present in 10% of all patients with cancer [73]; *RAF* mutations (particularly *B-RAF*) have been identified in about 8% of all cancer types [74]. The rate of extracellular signal-regulated kinase kinase (MEK) mutations is low (~1%), and only a few major pathogenic mutations in ERKs have been stated [53,71,72,73,74].

The RAF family (which includes A-RAF, B-RAF, and C-RAF or RAF1 gene isoforms) displays serine/threonine protein kinase activity after binding to RAS-guanosine triphosphate (GTP). Upon activation, C-RAF interacts with MEK and phosphorylates S218 and S222 residues of the MEK catalytic domain. Thus, when multiple kinases act on MEK, activated MEK directly catalyzes the phosphorylation of Tyr and Thr residues of ERKs, the last Ser/Thr protein kinase of the cascade. Once activated, ERK1/2 moves from the cytoplasm to the nucleus, influencing the activity of several transcription factors (such as proto-oncogenes c-FOS, c-JUN, ETS domain-containing protein ELK-1, c-MYC, cyclic AMP-dependent transcription factor ATF2, etc.), modulating cell cytoskeletal components such as microtubule-associated protein (MAP), cell metabolism, and cell fate. Many human malignancies have aberrant phosphorylation and activation of the downstream effector ERK1/2, suggesting that carcinogenesis depends on their activity [62,75]. B-RAF mutation occurs in 27–70% of malignant melanomas, 40–70% of papillary or anaplastic thyroid tumors, 30% of ovarian carcinomas, 5–22% of colorectal cancer, and a small percentages of carcinomas from other sites [76]. The most frequent B-RAF mutation entails thymine/adenine transversion at codon 600 (B-RAFV600E), with the replacement of a valine by glutamic acid into the polypeptide chain, causing an increase in its kinase activity. Interestingly, oncogenic ERK1/2 mutations have not yet been found, and activating ERK mutations has not been demonstrated to be a cause of cancer [77]. With a negative feedback regulatory loop, cytoplasmic ERK1/2 can phosphorylate other protein kinases upstream of the ERK pathway, such as SOS, C-RAF, and MEK [53]. Upstream to the RAF-MEK-ERK pathway is RAS. It was first unearthed as a family of viral oncogenes, H-RAS (Harvey’s Sarcoma), and K-RAS (Kirtsten’s Sarcoma) (another member, N-RAS, was discovered in neuroblastoma). Their products are small G proteins that after the stimulation of growth factors, cytokines or other activators (PKC, SRC, etc.) can switch from an active to an inactive conformation by swapping GTP and GDP, respectively, thus controlling signal transduction [77].

### 1.6. MAPK Signaling in Tumor Invasion and Metastasis

Through phosphorylating nuclear and cytoplasmic targets, the ERK/MAPK signaling pathway is also crucial for tumor invasion and metastasis. Accordingly, blocking the ERK/MAPK signaling pathway may inhibit the role of extracellular signals that promote cell movement, inhibiting tumor invasion and metastasis [78]. Experimental models using human ovarian cancer cells demonstrated that the activated ERK signaling pathway promoted the proliferation and migration of ovarian cancer ascites cells [28,79]. ERK activation can mediate tumor cell migratory activity by the up-regulation of genes involved in invasion, cell adhesion, extracellular matrix production, and degradation, and scaffold proteins that play essential roles in cell adhesion, transcription, and cytoskeletal organization [80,81,82] or matrix degradation [83,84]. Indeed, MEK inhibitors have been shown to block migration and invasion of many cancer cell types [85], including SK-OV-3 human ovarian carcinoma cells [86].

Downstream to activated ERKs is the ribosomal S6 kinases (p90-RSKs) family of kinases that has been involved in the regulation of tumor metastasis [80]. The RSK mechanism of action depends both on the isoform and the cancer type. Certain isoforms impact transcription and integrin activity to enhance cell motility and invasion, whereas other isoforms influence the actin cytoskeleton to hinder these processes [80]. However, despite the variance in RSK-mediated outcomes, chemical inhibition of this group of kinases has proven to be effective in blocking invasion and metastasis of several solid tumors in pre-clinical models [80].

### 1.7. Alterations of MAPK Pathway in TGCTs

Despite the fact that somatic mutations are rare in TGCTs, DNA exome sequencing has found three genes of relevance, KIT (18–25%), K-RAS (14%), and N-RAS (4%), all of which are exclusively detected in tumors with seminomatous components (either pure SE or as a component of mixed NSGCT) [87,88].

**Figure 2:**
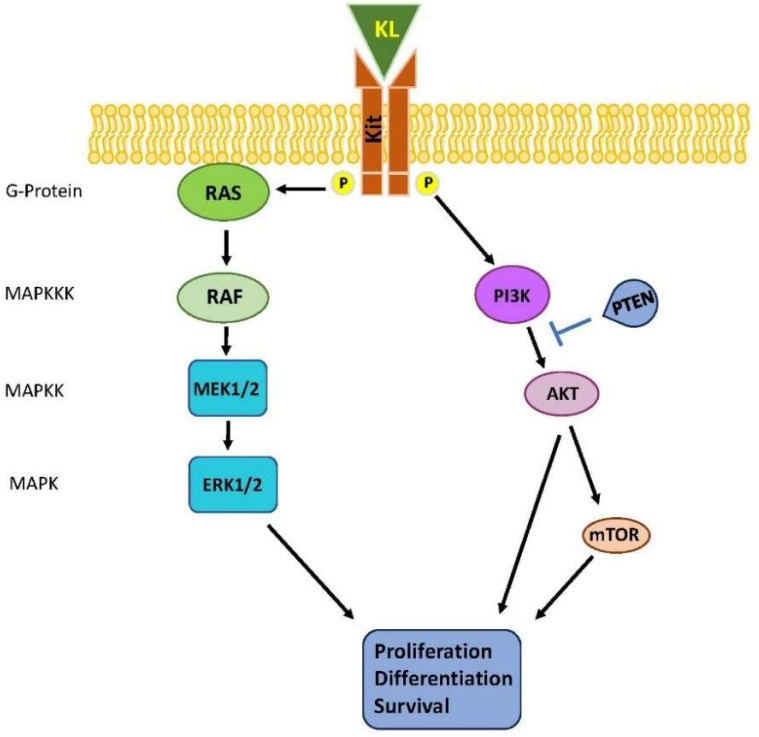
Schematic representation of deranged MAPK pathway in TGCTs. Arrows are activating stimuli, while blunt arrows indicate inhibitory stimuli.

*KIT* locus (chromosome 5) encodes for a type III receptor tyrosine kinase that dimerizes upon binding to its cognate ligand KL. Such an event leads to the activation of KIT intrinsic tyrosine kinase activity and the phosphorylation of key tyrosine residues within the receptor. The resulting phosphor–tyrosine residues serve as docking sites for molecules containing SH2 and other phosphor–tyrosine binding domains. Among the major signaling pathways activated by the KL/KIT, the RAS-RAF-MEK-ERK and PI3K/AKT pathways are involved in TGCTs (Figure 2).

Stimulation of KIT signaling through activating mutations and genomic amplification has been extensively demonstrated in TGCTs [88,89].

Activatory mutations reside within the activation loop of the kinase 2 domain, in the juxtamembrane domain, and in the protein tyrosine kinase 1 domain of the KIT gene. In an extended genomic analysis of a large cohort of patients affected by TGCTs, 8.1% of cases (28 SEs and 18 mixed/NSEs) resulted in somatic *KIT* mutations. Exons 17 (67.3%), 11 (22.4%), and 13 (6.1%) were the most affected exons. *KIT*-mutant cases were enriched for oncogenic RAS/MAPK pathway alterations compared to KIT-wildtype cases (34.8% vs. 19.2%, *p* = 0.02). Concurrent mutations in K-RAS (21.7%), RRAS2 (11.8%), CBL (6.5%), N-RAS (4.3%), MAP2K1 (2.2%), and RAC1 (2.2%) were seen among KIT-mutant individuals, while mutations in K-RAS, RRAS2, and N-RAS were mutually exclusive [90].

The *KL* locus maps to chromosome 12 in humans, and its gene product controls the survival and proliferation of both PGCs and postnatal male germ cells [9,24]. As previously anticipated, the *KL* locus acts as a modifier of TGCT susceptibility in the 129/Sv strain of mice mimicking pediatric TGCTs, human teratomas that develop from PGCs [21,91]. Indeed, among the most statistically significant mutated loci in TGCT patients is the *KL rs4474514* variant, which is associated with TGCT of the adolescent age [89,92].

Mutations of *K-RAS* and *N-RAS* account for about 26% and 4% of TGCTs, respectively, as reported from four studies in the *cBioportal* database [93]. The most recurrent *K-RAS* defects were amplifications and mutations at codon 12 [93,94]. *K-RAS* and *N-RAS* mutations were independently identified both in SEs and in NSEs. However, both mutations were also found to concomitantly occur in GCT patients carrying both SE and NSE components [95,96]. Accordingly, another study reported *RAS* mutations in 16% of SEs and in 15% of NSEs [97]. In an Indian TGCT patient set, the *K-RAS* mutation was identified in 17% of patients, the majority of whom were affected by mixed GCT (13%), while the remaining were represented by pure seminomas (4%). Metastatic tumors were shown to carry more *K-RAS* mutations than primary tumors (100% vs. 13%) [50]. *B-RAF* mutations are rare in TGCTs [93]. *B-RAF* missense mutations (1796T>A mutations) were found in 9% of NSEs within the EC component but not in SEs [96], while other studies did not detect any *B-RAF* defect in the TGCT cohorts studied [98,99]. *B-RAF* mutated GCTs were reported to occur at a high percentage (24%) within a chemotherapy refractoriness cohort showing platinum resistance and were reported to correlate to low or absent mismatch repair protein (MMR) expression and high microsatellite instability (MSI) [100]. These tumors are frequently associated with a mediastinal primary onset and with a trend to more prolonged progression-free survival [100]. In agreement, another study reported a positive correlation between MSI and the presence of a mutated *B-RAF* allele in ECs [96]. While *B-RAF* mutations were mutually exclusive with any *RAS* mutations [50], constitutively activated ERKs were detected in almost all tumors tested [96]. A summary of the mutated somatic genes in the cKIT–RAS–RAF–MEK–ERK cascade in TGCTs is reported in Table 1.

### 1.8. Prognosis and Response to Treatment

Cisplatin-based chemotherapy is the elective treatment for TGCT [102]. Previous reports have shown that cisplatin activates the MAPK pathway in several cancer types. However, it has not been established yet whether this activation is linked to apoptosis or to its prevention [103] and references therein]. Functional assays using MEK inhibitors indicated that MEK and ERK phosphorylation is necessary for caspase-3 activation by cisplatin either in *p53wt* or in *p53null* human teratocarcinoma cell lines [104]. Cisplatin resistance is determined by several biological mechanisms [27,105], among which the acquisition of DNA repair proficiency of cancer cells plays an important role in cisplatin-resistant TCGT cell lines [106]. Platinum refractoriness has been associated with the NSE subtypes and those that originate in extragonadal sites [107]. A recent study on the genomic landscape of platinum-resistant and -sensitive testicular cancers has shown that resistant tumors are enriched for somatic WNT/CTNNB1 pathway mutations that were detected in both platinum-resistant and metastatic TGCTs, while platinum-sensitive cancers were associated with mutated *KIT* alleles [88]. The RAS/RAF/MAPK-activated pathway plays an important role in this context, and kinase inhibitors that insist on this pathway can reverse platinum resistance in several cancer cell lines [108,109]. Interestingly, two independent studies reported the occurrence of *B-RAF* V600E mutations and MSI in unselected GCTs from cisplatin-resistant or relapsed NSE patient cohorts with an increased frequency [100,110].

## 2. Conclusions

Improved understanding of the genomic drivers of GCT development and heritability has the potential to yield valuable insight into GCT pathogenesis, helps patients at higher risk for GCT identification, and aids in the development of novel therapeutic approaches to benefit the cohort of patients with chemo-resistant disease. The RAS/RAF/MEK/ERK, as well as the KIT-activated pathways, have been demonstrated to be efficiently druggable, providing new therapy tools to limit tumor progression, particularly in patients that develop resistance to platinum-based treatments. However, despite the knowledge of this molecular pathway, no targeted therapies aimed at inhibiting MAPKs or KIT are approved for TGCT treatment.

Understanding the molecular mechanisms underlying GCT resistance to cisplatin is, therefore, essential to identify new targeted therapies.

## Figures and Tables

**Table 1:** Frequency of mutated somatic genes in the cKIT–RAS–RAF–MEK–ERK cascade in TGCTs.

		KIT	RAS	RAF	MEK/ERK
Sommerer et al., 2005 [96]	SE		7% 9%	0% 9%	Activated in all (but not mutated)
	NSE				
Solomon et al., 2011 [101]	SE	19% 2%	5–7% 0%	1% 2%	
	NSE				
Hacioglu et al., 2017 [97]	SE		29% 26%		
	NSE				
Shen et al., 2018 [90]	SE	18–25% 2%	KRAS:14% NRAS: 4%		
	NSE		low		
Ahmad et al., 2019 [50]	SE	0%	4% 31%	0%	
	NSE				

SE = seminomatous; NSE = mixed/Non-seminomatous.

## References

[1] Irie N., Weinberger L., Tang W.W., Kobayashi T., Viukov S., Manor Y.S., Dietmann S., Hanna J.H., Surani M.A. (2015). SOX17 is a critical specifier of human primordial germ cell fate. Cell.

[2] Tang W.W., Kobayashi T., Irie N., Dietmann S., Surani M.A. (2016). Specification and epigenetic programming of the human germ line. Nat. Rev. Genet..

[3] Chen D., Liu W., Zimmerman J., Pastor W.A., Kim R., Hosohama L., Ho J., Aslanyan M., Gell J.J., Jacobsen S.E. (2018). The TFAP2C-Regulated OCT4 Naive Enhancer Is Involved in Human Germline Formation. Cell Rep..

[4] Chen D., Sun N., Hou L., Kim R., Faith J., Aslanyan M., Tao Y., Zheng Y., Fu J., Liu W. (2019). Human Primordial Germ Cells Are Specified from Lineage-Primed Progenitors. Cell Rep..

[5] Freeman B. (2003). The active migration of germ cells in the embryos of mice and men is a myth. Reproduction.

[6] Gu Y., Runyan C., Shoemaker A., Surani A., Wylie C. (2009). Steel factor controls primordial germ cell survival and motility from the time of their specification in the allantois, and provides a continuous niche throughout their migration. Development.

[7] Oosterhuis J.W., Stoop H., Honecker F., Looijenga L.H. (2007). Why human extragonadal germ cell tumours occur in the midline of the body: Old concepts, new perspectives. Int. J. Androl..

[8] Shamblott M.J., Axelman J., Wang S., Bugg E.M., Littlefield J.W., Donovan P.J., Blumenthal P.D., Huggins G.R., Gearhart J.D. (1998). Derivation of pluripotent stem cells from cultured human primordial germ cells. Proc. Natl. Acad. Sci. USA.

[9] Dolci S., Williams D.E., Ernst M.K., Resnick J.L., Brannan C.I., Lock L.F., Lyman S.D., Boswell H.S., Donovan P.J. (1991). Requirement for mast cell growth factor for primordial germ cell survival in culture. Nature.

[10] Matsui Y., Zsebo K., Hogan B.L. (1992). Derivation of pluripotential embryonic stem cells from murine primordial germ cells in culture. Cell.

[11] Resnick J.L., Bixler L.S., Cheng L., Donovan P.J. (1992). Long-term proliferation of mouse primordial germ cells in culture. Nature.

[12] Donovan P.J., de Miguel M.P. (2003). Turning germ cells into stem cells. Curr. Opin. Genet. Dev..

[13] Conway A.E., Lindgren A., Galic Z., Pyle A.D., Wu H., Zack J.A., Pelligrini M., Teitell M.A., Clark A.T. (2009). A self-renewal program controls the expansion of genetically unstable cancer stem cells in pluripotent stem cell-derived tumors. Stem Cells.

[14] De Felici M., Dolci S. (2013). From testis to teratomas: A brief history of male germ cells in mammals. Int. J. Dev. Biol..

[15] Dolci S., Campolo F., De Felici M. (2015). Gonadal development and germ cell tumors in mouse and humans. Semin. Cell Dev. Biol..

[16] Oosterhuis J.W., Looijenga L.H. (2005). Testicular germ-cell tumours in a broader perspective. Nat. Rev. Cancer.

[17] Moch H., Cubilla A.L., Humphrey P.A., Reuter V.E., Ulbright T.M. (2016). The 2016 WHO Classification of Tumours of the Urinary System and Male Genital Organs-Part A: Renal, Penile, and Testicular Tumours. Eur. Urol..

[18] Reuter V.E. (2005). Origins and molecular biology of testicular germ cell tumors. Mod. Pathol..

[19] Skakkebaek N.E., Berthelsen J.G., Giwercman A., Muller J. (1987). Carcinoma-in-situ of the testis: Possible origin from gonocytes and precursor of all types of germ cell tumours except spermatocytoma. Int. J. Androl..

[20] Almstrup K., Sonne S.B., Hoei-Hansen C.E., Ottesen A.M., Nielsen J.E., Skakkebaek N.E., Leffers H., Rajpert-De Meyts E. (2006). From embryonic stem cells to testicular germ cell cancer—Should we be concerned?. Int. J. Androl..

[21] Guida E., Tassinari V., Colopi A., Todaro F., Cesarini V., Jannini B., Pellegrini M., Botti F., Rossi G., Rossi P. (2022). MAPK activation drives male and female mouse teratocarcinomas from late primordial germ cells. J. Cell Sci..

[22] Barchi M., Guida E., Dolci S., Rossi P., Grimaldi P. (2023). Endocannabinoid system and epigenetics in spermatogenesis and testicular cancer. Vitam. Horm..

[23] Tassinari V., Campolo F., Cesarini V., Todaro F., Dolci S., Rossi P. (2015). Fgf9 inhibition of meiotic differentiation in spermatogonia is mediated by Erk-dependent activation of Nodal-Smad2/3 signaling and is antagonized by Kit Ligand. Cell Death Dis..

[24] Dolci S., Pellegrini M., Di Agostino S., Geremia R., Rossi P. (2001). Signaling through extracellular signal-regulated kinase is required for spermatogonial proliferative response to stem cell factor. J. Biol. Chem..

[25] Di Siena S., Campolo F., Rossi P., Jannini E.A., Dolci S., Pellegrini M. (2013). UV and genotoxic stress induce ATR relocalization in mouse spermatocytes. Int. J. Dev. Biol..

[26] Litchfield K., Summersgill B., Yost S., Sultana R., Labreche K., Dudakia D., Renwick A., Seal S., Al-Saadi R., Broderick P. (2015). Whole-exome sequencing reveals the mutational spectrum of testicular germ cell tumours. Nat. Commun..

[27] Taylor-Weiner A., Zack T., O’Donnell E., Guerriero J.L., Bernard B., Reddy A., Han G.C., AlDubayan S., Amin-Mansour A., Schumacher S.E. (2016). Genomic evolution and chemoresistance in germ-cell tumours. Nature.

[28] Liu S.B., Lin X.P., Xu Y., Shen Z.F., Pan W.W. (2018). DAXX promotes ovarian cancer ascites cell proliferation and migration by activating the ERK signaling pathway. J. Ovarian Res..

[29] Martin F.C., Conduit C., Loveland K.L., Thomas B., Lewin J., Tran B. (2022). Genetics of testicular cancer: A review. Curr. Opin. Urol..

[30] Pluta J., Pyle L.C., Nead K.T., Wilf R., Li M., Mitra N., Weathers B., D’Andrea K., Almstrup K., Anson-Cartwright L. (2021). Identification of 22 susceptibility loci associated with testicular germ cell tumors. Nat. Commun..

[31] Krentz A.D., Murphy M.W., Zhang T., Sarver A.L., Jain S., Griswold M.D., Bardwell V.J., Zarkower D. (2013). Interaction between DMRT1 function and genetic background modulates signaling and pluripotency to control tumor susceptibility in the fetal germ line. Dev. Biol..

[32] Krentz A.D., Murphy M.W., Kim S., Cook M.S., Capel B., Zhu R., Matin A., Sarver A.L., Parker K.L., Griswold M.D. (2009). The DM domain protein DMRT1 is a dose-sensitive regulator of fetal germ cell proliferation and pluripotency. Proc. Natl. Acad. Sci. USA.

[33] Meissner A., Mikkelsen T.S., Gu H., Wernig M., Hanna J., Sivachenko A., Zhang X., Bernstein B.E., Nusbaum C., Jaffe D.B. (2008). Genome-scale DNA methylation maps of pluripotent and differentiated cells. Nature.

[34] Popp C., Dean W., Feng S., Cokus S.J., Andrews S., Pellegrini M., Jacobsen S.E., Reik W. (2010). Genome-wide erasure of DNA methylation in mouse primordial germ cells is affected by AID deficiency. Nature.

[35] Guo F., Yan L., Guo H., Li L., Hu B., Zhao Y., Yong J., Hu Y., Wang X., Wei Y. (2015). The Transcriptome and DNA Methylome Landscapes of Human Primordial Germ Cells. Cell.

[36] Sasaki H., Matsui Y. (2008). Epigenetic events in mammalian germ-cell development: Reprogramming and beyond. Nat. Rev. Genet..

[37] Hata K., Okano M., Lei H., Li E. (2002). Dnmt3L cooperates with the Dnmt3 family of de novo DNA methyltransferases to establish maternal imprints in mice. Development.

[38] Kota S.K., Feil R. (2010). Epigenetic transitions in germ cell development and meiosis. Dev. Cell.

[39] Kimmins S., Sassone-Corsi P. (2005). Chromatin remodelling and epigenetic features of germ cells. Nature.

[40] Messerschmidt D.M., Knowles B.B., Solter D. (2014). DNA methylation dynamics during epigenetic reprogramming in the germline and preimplantation embryos. Genes. Dev..

[41] Fendler A., Stephan C., Yousef G.M., Kristiansen G., Jung K. (2016). The translational potential of microRNAs as biofluid markers of urological tumours. Nat. Rev. Urol..

[42] Netto G.J., Nakai Y., Nakayama M., Jadallah S., Toubaji A., Nonomura N., Albadine R., Hicks J.L., Epstein J.I., Yegnasubramanian S. (2008). Global DNA hypomethylation in intratubular germ cell neoplasia and seminoma, but not in nonseminomatous male germ cell tumors. Mod. Pathol..

[43] Kristensen D.G., Nielsen J.E., Jorgensen A., Skakkebaek N.E., Rajpert-De Meyts E., Almstrup K. (2014). Evidence that active demethylation mechanisms maintain the genome of carcinoma in situ cells hypomethylated in the adult testis. Br. J. Cancer.

[44] Landero-Huerta D.A., Vigueras-Villasenor R.M., Yokoyama-Rebollar E., Arechaga-Ocampo E., Rojas-Castaneda J.C., Jimenez-Trejo F., Chavez-Saldana M. (2017). Epigenetic and risk factors of testicular germ cell tumors: A brief review. Front Biosci (Landmark Ed).

[45] Lister R., Pelizzola M., Dowen R.H., Hawkins R.D., Hon G., Tonti-Filippini J., Nery J.R., Lee L., Ye Z., Ngo Q.M. (2009). Human DNA methylomes at base resolution show widespread epigenomic differences. Nature.

[46] Shen Y., Zhang J., Liu Y., Liu S., Liu Z., Duan Z., Wang Z., Zhu B., Guo Y.L., Tian Z. (2018). DNA methylation footprints during soybean domestication and improvement. Genome Biol..

[47] Smiraglia D.J., Szymanska J., Kraggerud S.M., Lothe R.A., Peltomaki P., Plass C. (2002). Distinct epigenetic phenotypes in seminomatous and nonseminomatous testicular germ cell tumors. Oncogene.

[48] Burbee D.G., Forgacs E., Zochbauer-Muller S., Shivakumar L., Fong K., Gao B., Randle D., Kondo M., Virmani A., Bader S. (2001). Epigenetic inactivation of RASSF1A in lung and breast cancers and malignant phenotype suppression. J. Natl. Cancer Inst..

[49] Dammann R., Yang G., Pfeifer G.P. (2001). Hypermethylation of the cpG island of Ras association domain family 1A (RASSF1A), a putative tumor suppressor gene from the 3p21.3 locus, occurs in a large percentage of human breast cancers. Cancer Res..

[50] Ahmad F., Surve P., Natarajan S., Patil A., Pol S., Patole K., Das B.R. (2020). Aberrant epigenetic inactivation of RASSF1A and MGMT gene and genetic mutations of KRAS, cKIT and BRAF in Indian testicular germ cell tumours. Cancer Genet..

[51] Ghasemi M., Samaei N.M., Mowla S.J., Shafiee M., Vasei M., Ghasemian N. (2018). Upregulation of miR-371-373 cluster, a human embryonic stem cell specific microRNA cluster, in esophageal squamous cell carcinoma. J. Cancer Res. Ther..

[52] Das M.K., Evensen H.S.F., Furu K., Haugen T.B. (2019). miRNA-302s may act as oncogenes in human testicular germ cell tumours. Sci. Rep..

[53] Guo Y.J., Pan W.W., Liu S.B., Shen Z.F., Xu Y., Hu L.L. (2020). ERK/MAPK signalling pathway and tumorigenesis. Exp. Ther. Med..

[54] Wang X., Tournier C. (2006). Regulation of cellular functions by the ERK5 signalling pathway. Cell Signal.

[55] Kolch W., Calder M., Gilbert D. (2005). When kinases meet mathematics: The systems biology of MAPK signalling. FEBS Lett..

[56] Kolch W. (2005). Coordinating ERK/MAPK signalling through scaffolds and inhibitors. Nat. Rev. Mol. Cell Biol..

[57] Kyriakis J.M., Avruch J. (2001). Mammalian mitogen-activated protein kinase signal transduction pathways activated by stress and inflammation. Physiol. Rev..

[58] Chang L., Karin M. (2001). Mammalian MAP kinase signalling cascades. Nature.

[59] Songyang Z., Lu K.P., Kwon Y.T., Tsai L.H., Filhol O., Cochet C., Brickey D.A., Soderling T.R., Bartleson C., Graves D.J. (1996). A structural basis for substrate specificities of protein Ser/Thr kinases: Primary sequence preference of casein kinases I and II, NIMA, phosphorylase kinase, calmodulin-dependent kinase II, CDK5, and Erk1. Mol. Cell Biol..

[60] Chen Z., Gibson T.B., Robinson F., Silvestro L., Pearson G., Xu B., Wright A., Vanderbilt C., Cobb M.H. (2001). MAP kinases. Chem. Rev..

[61] Porter A.C., Vaillancourt R.R. (1998). Tyrosine kinase receptor-activated signal transduction pathways which lead to oncogenesis. Oncogene.

[62] Blume-Jensen P., Hunter T. (2001). Oncogenic kinase signalling. Nature.

[63] Yarden Y. (2001). The EGFR family and its ligands in human cancer. signalling mechanisms and therapeutic opportunities. Eur. J. Cancer.

[64] Bache K.G., Slagsvold T., Stenmark H. (2004). Defective downregulation of receptor tyrosine kinases in cancer. EMBO J..

[65] Hanahan D., Weinberg R.A. (2011). Hallmarks of cancer: The next generation. Cell.

[66] Zandi R., Larsen A.B., Andersen P., Stockhausen M.T., Poulsen H.S. (2007). Mechanisms for oncogenic activation of the epidermal growth factor receptor. Cell Signal.

[67] Klein S., Levitzki A. (2009). Targeting the EGFR and the PKB pathway in cancer. Curr. Opin. Cell Biol..

[68] Karnoub A.E., Weinberg R.A. (2008). Ras oncogenes: Split personalities. Nat. Rev. Mol. Cell Biol..

[69] Bollag G., Tsai J., Zhang J., Zhang C., Ibrahim P., Nolop K., Hirth P. (2012). Vemurafenib: The first drug approved for BRAF-mutant cancer. Nat. Rev. Drug Discov..

[70] Garcia-Gomez R., Bustelo X.R., Crespo P. (2018). Protein-Protein Interactions: Emerging Oncotargets in the RAS-ERK Pathway. Trends Cancer.

[71] Khotskaya Y.B., Holla V.R., Farago A.F., Mills Shaw K.R., Meric-Bernstam F., Hong D.S. (2017). Targeting TRK family proteins in cancer. Pharmacol. Ther..

[72] Maik-Rachline G., Hacohen-Lev-Ran A., Seger R. (2019). Nuclear ERK: Mechanism of Translocation, Substrates, and Role in Cancer. Int. J. Mol. Sci..

[73] Sanchez-Vega F., Mina M., Armenia J., Chatila W.K., Luna A., La K.C., Dimitriadoy S., Liu D.L., Kantheti H.S., Saghafinia S. (2018). Oncogenic Signaling Pathways in The Cancer Genome Atlas. Cell.

[74] Holderfield M., Deuker M.M., McCormick F., McMahon M. (2014). Targeting RAF kinases for cancer therapy: BRAF-mutated melanoma and beyond. Nat. Rev. Cancer.

[75] Kohno M., Pouyssegur J. (2006). Targeting the ERK signaling pathway in cancer therapy. Ann. Med..

[76] Davies H., Bignell G.R., Cox C., Stephens P., Edkins S., Clegg S., Teague J., Woffendin H., Garnett M.J., Bottomley W. (2002). Mutations of the BRAF gene in human cancer. Nature.

[77] Terrell E.M., Morrison D.K. (2019). Ras-Mediated Activation of the Raf Family Kinases. Cold Spring Harb. Perspect. Med..

[78] Burotto M., Chiou V.L., Lee J.M., Kohn E.C. (2014). The MAPK pathway across different malignancies: A new perspective. Cancer.

[79] Zhao J., Ye W., Wu J., Liu L., Yang L., Gao L., Chen B., Zhang F., Yang H., Li Y. (2015). Sp1-CD147 positive feedback loop promotes the invasion ability of ovarian cancer. Oncol. Rep..

[80] Sulzmaier F.J., Ramos J.W. (2013). RSK isoforms in cancer cell invasion and metastasis. Cancer Res..

[81] Gialeli C., Theocharis A.D., Karamanos N.K. (2011). Roles of matrix metalloproteinases in cancer progression and their pharmacological targeting. FEBS J..

[82] Maeda-Yamamoto M., Suzuki N., Sawai Y., Miyase T., Sano M., Hashimoto-Ohta A., Isemura M. (2003). Association of suppression of extracellular signal-regulated kinase phosphorylation by epigallocatechin gallate with the reduction of matrix metalloproteinase activities in human fibrosarcoma HT1080 cells. J. Agric. Food Chem..

[83] Braicu C., Buse M., Busuioc C., Drula R., Gulei D., Raduly L., Rusu A., Irimie A., Atanasov A.G., Slaby O. (2019). A Comprehensive Review on MAPK: A Promising Therapeutic Target in Cancer. Cancers.

[84] Gao J., Wang Y., Yang J., Zhang W., Meng K., Sun Y., Li Y., He Q.Y. (2019). RNF128 Promotes Invasion and Metastasis via the EGFR/MAPK/MMP-2 Pathway in Esophageal Squamous Cell Carcinoma. Cancers.

[85] Horiuchi H., Kawamata H., Furihata T., Omotehara F., Hori H., Shinagawa Y., Ohkura Y., Tachibana M., Yamazaki T., Ajiki T. (2004). A MEK inhibitor (U0126) markedly inhibits direct liver invasion of orthotopically inoculated human gallbladder cancer cells in nude mice. J. Exp. Clin. Cancer Res..

[86] Basu M., Mukhopadhyay S., Chatterjee U., Roy S.S. (2014). FGF16 promotes invasive behavior of SKOV-3 ovarian cancer cells through activation of mitogen-activated protein kinase (MAPK) signaling pathway. J. Biol. Chem..

[87] Todaro F., Campolo F., Barrios F., Pellegrini M., Di Cesare S., Tessarollo L., Rossi P., Jannini E.A., Dolci S. (2019). Regulation of Kit Expression in Early Mouse Embryos and ES Cells. Stem Cells.

[88] Loveday C., Litchfield K., Proszek P.Z., Cornish A.J., Santo F., Levy M., Macintyre G., Holryod A., Broderick P., Dudakia D. (2020). Genomic landscape of platinum resistant and sensitive testicular cancers. Nat. Commun..

[89] McIntyre A., Summersgill B., Grygalewicz B., Gillis A.J., Stoop J., van Gurp R.J., Dennis N., Fisher C., Huddart R., Cooper C. (2005). Amplification and overexpression of the KIT gene is associated with progression in the seminoma subtype of testicular germ cell tumors of adolescents and adults. Cancer Res..

[90] Shen H., Shih J., Hollern D.P., Wang L., Bowlby R., Tickoo S.K., Thorsson V., Mungall A.J., Newton Y., Hegde A.M. (2018). Integrated Molecular Characterization of Testicular Germ Cell Tumors. Cell Rep..

[91] Pierpont T.M., Lyndaker A.M., Anderson C.M., Jin Q., Moore E.S., Roden J.L., Braxton A., Bagepalli L., Kataria N., Hu H.Z. (2017). Chemotherapy-Induced Depletion of OCT4-Positive Cancer Stem Cells in a Mouse Model of Malignant Testicular Cancer. Cell Rep..

[92] Poynter J.N., Hooten A.J., Frazier A.L., Ross J.A. (2012). Associations between variants in KITLG, SPRY4, BAK1, and DMRT1 and pediatric germ cell tumors. Genes. Chromosomes Cancer.

[93] Cerami E., Gao J., Dogrusoz U., Gross B.E., Sumer S.O., Aksoy B.A., Jacobsen A., Byrne C.J., Heuer M.L., Larsson E. (2012). The cBio cancer genomics portal: An open platform for exploring multidimensional cancer genomics data. Cancer Discov..

[94] Roelofs H., Mostert M.C., Pompe K., Zafarana G., van Oorschot M., van Gurp R.J., Gillis A.J., Stoop H., Beverloo B., Oosterhuis J.W. (2000). Restricted 12p amplification and RAS mutation in human germ cell tumors of the adult testis. Am. J. Pathol..

[95] Boublikova L., Bakardjieva-Mihaylova V., Skvarova Kramarzova K., Kuzilkova D., Dobiasova A., Fiser K., Stuchly J., Kotrova M., Buchler T., Dusek P. (2016). Wilms tumor gene 1 (WT1), TP53, RAS/BRAF and KIT aberrations in testicular germ cell tumors. Cancer Lett..

[96] Sommerer F., Hengge U.R., Markwarth A., Vomschloss S., Stolzenburg J.U., Wittekind C., Tannapfel A. (2005). Mutations of BRAF and RAS are rare events in germ cell tumours. Int. J. Cancer.

[97] Hacioglu B.M., Kodaz H., Erdogan B., Cinkaya A., Tastekin E., Hacibekiroglu I., Turkmen E., Kostek O., Genc E., Uzunoglu S. (2017). K-RAS and N-RAS mutations in testicular germ cell tumors. Bosn. J. Basic. Med. Sci..

[98] McIntyre A., Summersgill B., Spendlove H.E., Huddart R., Houlston R., Shipley J. (2005). Activating mutations and/or expression levels of tyrosine kinase receptors GRB7, RAS, and BRAF in testicular germ cell tumors. Neoplasia.

[99] Carcano F.M., Lengert A.H., Vidal D.O., Scapulatempo Neto C., Queiroz L., Marques H., Baltazar F., Berardinelli G.N., Martinelli C.M., da Silva E.C. (2016). Absence of microsatellite instability and BRAF (V600E) mutation in testicular germ cell tumors. Andrology.

[100] Honecker F., Wermann H., Mayer F., Gillis A.J., Stoop H., van Gurp R.J., Oechsle K., Steyerberg E., Hartmann J.T., Dinjens W.N. (2009). Microsatellite instability, mismatch repair deficiency, and BRAF mutation in treatment-resistant germ cell tumors. J. Clin. Oncol..

[101] Solomon H., Madar S., Rotter V. (2011). Mutant p53 gain of function is interwoven into the hallmarks of cancer. J. Pathol..

[102] Einhorn L.H., Donohue J. (1977). Cis-diamminedichloroplatinum, vinblastine, and bleomycin combination chemotherapy in disseminated testicular cancer. Ann. Intern. Med..

[103] Achkar I.W., Abdulrahman N., Al-Sulaiti H., Joseph J.M., Uddin S., Mraiche F. (2018). Cisplatin based therapy: The role of the mitogen activated protein kinase signaling pathway. J. Transl. Med..

[104] Schweyer S., Soruri A., Meschter O., Heintze A., Zschunke F., Miosge N., Thelen P., Schlott T., Radzun H.J., Fayyazi A. (2004). Cisplatin-induced apoptosis in human malignant testicular germ cell lines depends on MEK/ERK activation. Br. J. Cancer.

[105] Chen S.H., Chang J.Y. (2019). New Insights into Mechanisms of Cisplatin Resistance: From Tumor Cell to Microenvironment. Int. J. Mol. Sci..

[106] Caggiano C., Cavallo F., Giannattasio T., Cappelletti G., Rossi P., Grimaldi P., Feldman D.R., Jasin M., Barchi M. (2021). Testicular Germ Cell Tumors Acquire Cisplatin Resistance by Rebalancing the Usage of DNA Repair Pathways. Cancers.

[107] Bokemeyer C., Nichols C.R., Droz J.P., Schmoll H.J., Horwich A., Gerl A., Fossa S.D., Beyer J., Pont J., Kanz L. (2002). Extragonadal germ cell tumors of the mediastinum and retroperitoneum: Results from an international analysis. J. Clin. Oncol..

[108] Jin L., Chun J., Pan C., Li D., Lin R., Alesi G.N., Wang X., Kang H.B., Song L., Wang D. (2018). MAST1 Drives Cisplatin Resistance in Human Cancers by Rewiring cRaf-Independent MEK Activation. Cancer Cell.

[109] Kong L.R., Chua K.N., Sim W.J., Ng H.C., Bi C., Ho J., Nga M.E., Pang Y.H., Ong W.R., Soo R.A. (2015). MEK Inhibition Overcomes Cisplatin Resistance Conferred by SOS/MAPK Pathway Activation in Squamous Cell Carcinoma. Mol. Cancer Ther..

[110] Mayer F., Wermann H., Albers P., Stoop H., Gillis A.J., Hartmann J.T., Bokemeyer C.C., Oosterhuis J.W., Looijenga L.H., Honecker F. (2011). Histopathological and molecular features of late relapses in non-seminomas. BJU Int..

